# Tumor Rupture in a Patient with Recurrent Hepatocellular Carcinoma Following Atezolizumab plus Bevacizumab: A Case Report

**DOI:** 10.70352/scrj.cr.25-0331

**Published:** 2025-07-17

**Authors:** Kazuhiro Tada, Shohei Yoshiya, Takaaki Mori, Ryoichi Narita, Kentaro Iwaki, Sota Nakamura, Yasunari Yamada, Hajime Takaki, Yo-ichi Yamashita, Kengo Fukuzawa

**Affiliations:** 1 Department of Surgery, Oita Red Cross Hospital, Oita, Oita, Japan; 2 Department of Radiology, Oita Red Cross Hospital, Oita, Oita, Japan; 3 Department of Hepato Biliary Pancreatic Internal Medicine, Oita Red Cross Hospital, Oita, Oita, Japan

**Keywords:** atezolizumab plus bevacizumab, hepatocellular carcinoma, rupture

## Abstract

**INTRODUCTION:**

Atezolizumab plus bevacizumab combination therapy (atezo-bev) has been widely used worldwide as the first-line treatment for unresectable hepatocellular carcinoma (HCC). Intratumoral hemorrhage has been reported as a rare adverse event of atezo-bev. However, no previous cases of HCC rupture during atezo-bev have been reported. Herein, we demonstrate a case of tumor rupture in a patient with recurrent HCC following atezo-bev, who was successfully managed with transcatheter arterial embolization (TAE).

**CASE PRESENTATION:**

An 80-year-old man with multiple recurrent HCCs (at least 4 lesions with a maximum tumor diameter of 35 mm protruding from the liver surface) developed epigastralgia immediately after the first cycle of atezo-bev. CT revealed a ruptured HCC in the left lateral lobe. TAE was performed, and the patient continued to receive atezo-bev without any subsequent tumor bleeding.

**CONCLUSIONS:**

Tumor rupture should be considered in patients with HCC following atezo-bev treatment.

## Abbreviations

atezo-bev
: atezolizumab plus bevacizumab therapy
HCC
: hepatocellular carcinoma
ICI
: immune checkpoint inhibitor
TACE
: transcatheter arterial chemoembolization
TAE
: transcatheter arterial embolization
VEGF
: vascular endothelial growth factor

## INTRODUCTION

The global phase 3 trial (IMbrave150) demonstrated that atezolizumab plus bevacizumab combination therapy (atezo-bev) had greater benefits than did sorafenib therapy in patients with unresectable hepatocellular carcinoma (HCC).^1)^ Since then, atezo-bev has been recommended as the first-line treatment for advanced HCC.^2)^ However, several adverse events associated with this combination therapy have also been reported. Bleeding is a well-known adverse reaction of bevacizumab.^3)^ The incidence of bleeding events was reported to be 7% in patients receiving atezo-bev compared to 4.5% in those receiving sorafenib therapy.^1)^ Although gastrointestinal bleeding is a common complication, atezo-bev-induced tumor bleeding is relatively rare. Moreover, no previous study has reported tumor rupture during atezo-bev therapy.

Herein, we report a case of tumor rupture in a patient with recurrent HCC following atezo-bev, which was successfully managed with transcatheter arterial embolization (TAE) using gelatin sponge particles.

## CASE PRESENTATION

**Fig. 1:**
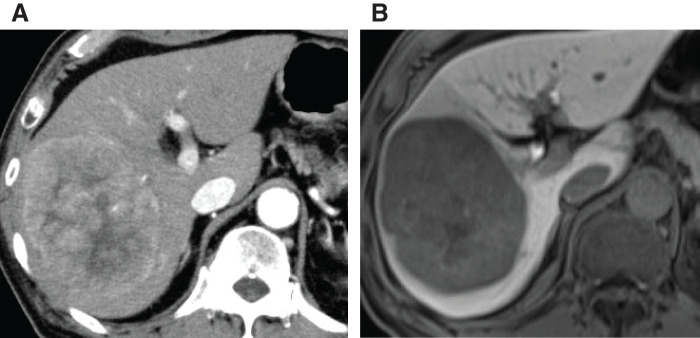
Abdominal contrast-enhanced CT (**A**) and MRI (**B**) findings before operation. (**A**) An enhanced mass measuring 97 × 78 mm in diameter is identified in the right lobe of the liver on arterial-phase imaging. (**B**) A large mass measuring 100 × 80 mm in diameter is observed in the right lobe of the liver, and the tumor boundary appears clear on the hepatobiliary phase image.

**Fig. 2:**
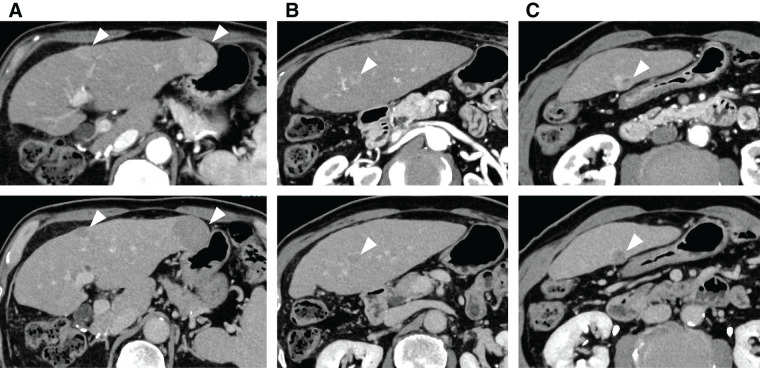
Abdominal contrast-enhanced CT images taken before atezolizumab plus bevacizumab therapy (above: arterial phase images; below: equilibrium phase images). (**A**) A 14-mm HCC in S3 and a 35-mm HCC lateral to S2 are detected. (**B**) A 10-mm HCC in the middle part of S2 is detected. (**C**) A 13-mm HCC in the caudal part of S2 is detected. Four masses are identified in the lateral segment of the liver, showing hyperenhancement in the arterial phase (above images in **A**–**C**) and washout in the equilibrium phase (below images in **A**–**C**), and are diagnosed as HCCs. HCC, hepatocellular carcinoma

**Fig. 3:**
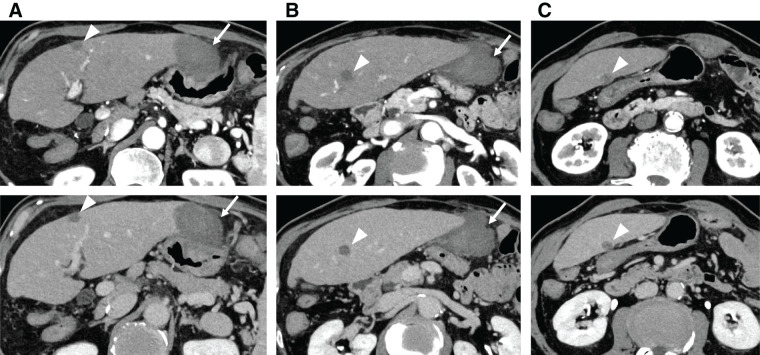
Abdominal contrast-enhanced CT images taken after the first cycle of atezolizumab plus bevacizumab therapy (above: arterial phase images; below: equilibrium phase images). No changes are observed in tumor size (arrowheads); however, the vascularity of all tumors is markedly suppressed (**A**–**C**). Ruptured HCC is identified in the lateral portion of S2 (arrows); however, the hematoma is localized in the subcapsular region, and no extravasation or pseudoaneurysm is observed (**A**, **B**). HCC, hepatocellular carcinoma

**Fig. 4:**
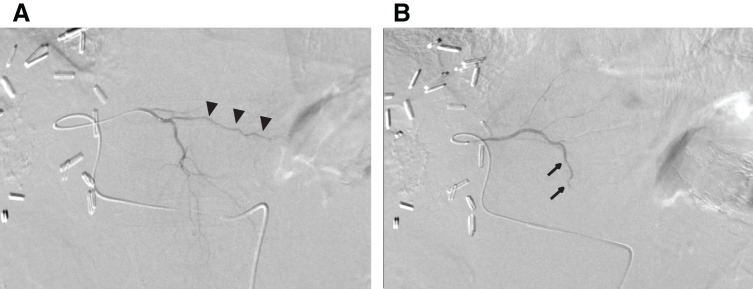
Transcatheter arterial embolization. (**A**) Angiography of the A2–A3 common vessel reveals no tumor staining. The A2 vessel is indicated by arrowheads. (**B**) After arterial embolization of A2, the A2 vessel is no longer visible, and the patency of the A3 vessel is preserved. The A3 vessel is indicated by the arrows.

An 80-year-old man with a history of alcoholic hepatitis underwent a laparoscopic right hemihepatectomy for a large HCC (**Fig. 1**). Histological examination of the resected specimen revealed a moderately to poorly differentiated simple nodular HCC with extranodal growth. The microvascular invasion statuses were vp0, vv1, and va0. No intrahepatic metastases or bile duct infiltration were observed. Six months later, contrast-enhanced CT scans revealed multiple recurrent HCCs in the left lobe (**Fig. 2**). Liver function was well preserved, categorized as Child–Pugh class A (6 points) and ALBI (albumin-bilirubin) score −1.80 (modified ALBI grade 2b), and the patient’s performance status was zero (**Table 1**). He had been prescribed medications for diabetes mellitus and hypertension, but had never received anticoagulants. We offered the patient transcatheter arterial chemoembolization (TACE) and systemic drug therapy; the patient preferred drug therapy. He experienced epigastric discomfort 4 days after the initiation of atezo-bev, but did not visit the hospital. The patient visited our hospital on the day of the second atezo-bev cycle. The epigastric discomfort had improved, but the serum C-reactive protein level was elevated (4.4 mg/dL). Abdominal ultrasound sonography indicated an echo-free space in the left upper abdomen. A contrast-enhanced CT scan showed a ruptured tumor at the edge of the left lateral lobe without extravasation, and the vascularity of all tumors was markedly suppressed (**Fig. 3**). A nonenhanced CT demonstrated high-attenuation areas around the subcapsule of the tumor in the left lateral lobe; moreover, the attenuation value was high in the pelvic fluid collection, indicating intra-abdominal bleeding as a result of the HCC rupture. Since the patient’s vital signs and general condition were stable and there was no anemia (**Table 1**), elective TAE was performed 3 days after the visit. The primary purpose of TAE was to prevent tumor rebleeding after the resumption of atezo-bev. There was no evidence of overt tumor staining on angiography, indicating a significant decrease in tumor vascularity. The feeder of the ruptured tumor was assumed to be the A2 vessel; therefore, we embolized the A2 vessel using gelatin sponge particles (**Fig. 4**). Following treatment, there were no adverse events and no change in hepatic reserve (**Table 1**), and the patient was discharged 6 days after TAE. We determined that atezo-bev was highly effective and decided to continue it. After TAE, atezo-bev was resumed, and no tumor bleeding has been noted since then.

**Table 1:** Blood examination results before and after TAE

	Before TAE	After TAE
WBC (/mm^3^)	6200	6800
RBC (/mm^3^)	426 × 10^4^	425 × 10^4^
Hemoglobin (g/dL)	13.5	13.4
Hematocrit (%)	40.2	40.3
Platelets (/mm^3^)	16.3 × 10^4^	13.3 × 10^4^
AST (U/L)	39	43
ALT (U/L)	30	32
LDH (U/L)	304	275
GGT (U/L)	198	179
ALP (U/L)	135	135
Total bilirubin (mg/dL)	0.8	0.8
BUN (mg/dL)	12	13
Creatinine (mg/dL)	0.66	0.6
CRP (mg/dL)	4.4	2.6
Alb (g/dL)	3.0	2.8
PT (%)	80.3	78.8
ALBI score	−1.80	−1.63
HBs antigen	Negative	—
Anti-HCV (S/CO)	0.04	—

Alb, albumin; ALBI, albumin-bilirubin; ALP, alkaline phosphatase; ALT, alanine transaminase; AST, aspartate transaminase; BUN, blood urea nitrogen; CRP, C-reactive protein; GGT, γ-glutamyl transpeptidase; HB, hepatitis B; HCV, hepatitis C virus; LDH, lactate dehydrogenase; PT, prothrombin time; RBC, red blood cell; S/CO, signal-to-cutoff ratio; WBC, white blood cell

## DISCUSSION

To our knowledge, this is the first report of tumor rupture in a patient with unresectable HCC following atezo-bev treatment that was successfully managed with TAE therapy using gelatin sponge particles. Previous reports have described intratumoral hemorrhage in HCC during atezo-bev (**Table 2**).^4,5)^ Bevacizumab is a humanized monoclonal antibody against vascular endothelial growth factor (VEGF), and its anti-VEGF effect is related to hemorrhagic risk.^6)^ Ramucirumab, a VEGF receptor-2-targeting human immunoglobulin G monoclonal antibody, is also a therapeutic option for HCC. In the REACH-2 trial, the incidences of grade 3 or higher bleeding events were 5.1% and 3.2% in the ramucirumab and placebo groups, respectively.^7)^ So far, only one case of ramucirumab-induced HCC rupture has been reported, but there is concern regarding serious antiangiogenic adverse events during anti-VEGF drug treatment.^8)^ Lenvatinib, an inhibitor of multiple receptor tyrosine kinases, including VEGF receptors, has a pharmaceutical effect similar to that of bevacizumab, and intratumoral hemorrhage and rupture of HCC due to lenvatinib have been reported.^9–11)^ Uchida-Kobayashi et al. reported that 5 (7.4%) of the 68 consecutive patients who received lenvatinib treatment in their retrospective single-center study developed tumor hemorrhage (including 2 cases of rupture). Interestingly, hemorrhage occurred immediately after initiating lenvatinib treatment, with an average dosing period of 4.4 days.^9)^ The mechanism underlying HCC rupture is considered to be the antitumoral effect of lenvatinib, which inhibits tumor angiogenesis and causes the remaining poorly developed tumor microvasculature to collapse.^12)^

**Table 2:** Previous reports of intratumoral hemorrhage related to atezolizumab plus bevacizumab in patients with HCC

Reference	Year	Symptom	Bleeding point	Rupture	Treatment for hemorrhage	Prognosis	Follow-up treatment
Mitsuyama et al.^4)^	2023	Fatigue	Rib metastasis from HCC	No	TAE	Survived	Atezo-bev
Park et al.^5)^	2024	Abdominal pain	Intratumoral hemorrhage	No	TAE	Died due to liver failure	–
Our case	2025	Epigastric discomfort	Intratumoral hemorrhage	Yes	TAE	Survived	Atezo-bev

Atezo-bev, atezolizumab-bevacizumab; HCC, hepatocellular carcinoma; TAE, transcatheter arterial embolization

Spontaneous HCC rupture occasionally occurs, with an incidence of 2.3%–26%.^13)^ Although the mechanism underlying HCC rupture has not been fully elucidated, several hypotheses have been proposed. According to the intratumoral pressure hypothesis, rapid tumor growth, tumor necrosis, and occlusion of the hepatic vein caused by tumor invasion are related to an increase in the internal pressure of the tumor.^12–14)^ Several studies have reported that tumor size is greater in ruptured HCC than in nonruptured HCC.^9,15,16)^ Previous research has reported that HCCs >5 cm are at high risk of tumor rupture.^16,17)^ Tumor protrusion from the original liver parenchyma may also be related to tumor rupture.^16)^ Li et al. reported that tumor location is related to rupture, and that the majority of ruptured HCCs are located in the left lateral segments (S2 and S3) and the right posterior-inferior segment (S6).^18)^ Sorafenib and lenvatinib, which are multikinase inhibitors of the VEGF pathway, may increase the risk of bleeding and HCC rupture in susceptible individuals.^9,12,13)^ Anti-VEGF drugs, including bevacizumab, can cause the small arteries supplying the tumor to become fragile. Atezolizumab, an immune checkpoint inhibitor (ICI) that exerts antitumor effects by modulating the immune function, may also contribute to HCC rupture. ICIs may trigger “pseudoprogression,” in which a tumor grows before it shrinks.^19)^ Pseudoprogression is thought to be caused by the infiltration of immune cells, followed by tumor necrosis, hemorrhage, and edema in lesions. To date, only a few cases of pseudoprogression in HCC have been reported^20,21)^; however, there is concern regarding pseudoprogression-induced HCC rupture. In our case, the risk of HCC rupture may have been relatively high because of tumor protrusion from the liver surface in segment 2. However, tumor rupture occurred 4 days after atezo-bev administration, and CT findings revealed remarkable suppression of vascularity with a dramatic decline in tumor markers (**Table 3**); therefore, we speculated that atezo-bev triggered the HCC rupture. In cases of HCC rupture, TAE plays a crucial role in achieving hemostasis and stabilizing the patient's condition. Emergency hepatectomy can be performed in some cases; however, in many cases, patients with ruptured HCC do not tolerate laparotomy well.^14)^

**Table 3:** Changes in tumor markers

	Before atezo-bev	After 1st cycle of atezo-bev
AFP (ng/mL)	2824	159
DCP (mAU/mL)	42.9	17.5

AFP, α-fetoprotein; DCP, des-γ-carboxyprothrombin

Recently, atezo-bev therapy followed by curative conversion has been demonstrated for the treatment of unresectable, intermediate-stage HCC. Atezo-bev followed by TACE achieved a higher complete response rate than atezo-bev therapy alone.^22)^ Atezo-bev exerts anti-VEGF effects before TACE, thereby enhancing the effects of TACE.^22)^ There are various situations among intermediate-stage HCC, and the indications and timing for attempting conversion to curative therapy should be carefully considered. According to previous reports, HCC rupture often occurs soon after the administration of anti-VEGF agents.^9–11)^ When planning to administer atezo-bev to patients with HCC located on the liver surface, as in our case, TAE or TACE before the initiation of atezo-bev might be effective in preventing HCC rupture. Furthermore, removing only the HCC with a risk of rupture in advance using laparoscopic partial hepatectomy may safely allow systemic chemotherapy for the remaining HCCs.

## CONCLUSIONS

The findings of this case suggest that clinicians should consider tumor rupture and check the tumor blood flow when abdominal pain appears after the initiation of atezo-bev for HCC in patients with risk factors for rupture.

## ACKNOWLEDGMENTS

We would like to thank Editage (www.editage.jp) for English language editing.

## DECLARATIONS

### Funding

No financial support was provided.

### Authors’ contributions

KT drafted the manuscript.

KT, SY, KI, and SN were involved in patient care.

TM, YYamada, and HT performed the interventional radiology procedures.

SY, RN, YYamada, and YYamashita contributed to manuscript review and editing.

KF gave final approval for the publication version.

All authors read and approved the final manuscript.

### Availability of data and materials

All data generated during this study are included in this article.

### Ethics approval and consent to participate

Not applicable.

### Consent for publication

Written informed consent was obtained from the patient for publication of this case report and any accompanying images.

### Competing interests

The authors declare that they have no competing interests.
